# ﻿Three new species of *Velia* (*Cesavelia*) Koçak & Kemal, 2010 (Hemiptera, Heteroptera, Veliidae) from China

**DOI:** 10.3897/zookeys.1254.156152

**Published:** 2025-10-03

**Authors:** Siying Fu, Zezhong Jin, Zhen Ye

**Affiliations:** 1 Institute of Entomology, College of Life Sciences, Nankai University, Tianjin, 300071, China Nankai University Tianjin China

**Keywords:** Hengduan Mountains, Himalayas, identification key, new species, riffle bugs, taxonomy, Veliidae, water striders

## Abstract

Velia (Cesavelia) lii**sp. nov.** and Velia (Cesavelia) motuoensis**sp. nov.** from Xizang Province, China, as well as Velia (Cesavelia) yiliangensis**sp. nov.** from Yunnan Province, China, are described. Photographs illustrating the habitus in dorsal and lateral views, male metafemora, genitalic structures, and habitats, as well as a key to the species of Velia (Cesavelia) are provided, along with an updated distribution map of the subgenus.

## ﻿Introduction

The genus *Velia* Latreille, 1804, is currently divided into three subgenera, each characterized by allopatric distributions. The subgenus Velia Latreille, 1804, comprises a single extant species, which is distributed across the western Mediterranean (Tamanini 1947; Andersen 1995; Berchi et al. 2018). The subgenus Plesiovelia Tamanini, 1955, includes 28 taxa (23 species and 5 subspecies), distributed from western Europe to northwestern India, with an extension into northern Africa (Andersen 1981, 1995; Tran et al. 2009; Berchi et al. 2018; Jin et al. 2023). The subgenus Cesavelia Koçak & Kemal, 2010, contains 11 species distributed across the Oriental Region, including northern India, Nepal, central and southern China, and northern Vietnam (Andersen 1981; J. Polhemus and D. Polhemus, 1999; D. Polhemus and J. Polhemus 2003; Tran et al. 2009; Koçak and Kemal 2010; Basu et al. 2013). *Cesavelia* exhibits high species diversity along the southern slopes of the Himalayas and the Hengduan Mountains (Tran et al. 2009; Jin et al. 2023). Ongoing exploration of the region has led to the discovery and description of additional new species. In the present study, we describe three new species: Velia (Cesavelia) lii sp. nov. and Velia (Cesavelia) motuoensis sp. nov. from Xizang Province, China, and Velia (Cesavelia) yiliangensis sp. nov. from Yunnan Province, China. These findings contribute further insight into the diversity patterns of *Velia* in this region. Additionally, this paper provides photographs of the habitus in dorsal and lateral views, male metafemora, genitalic structures, and habitats of new species described in this study, along with a distribution map of described species of *Cesavelia* to date.

## ﻿Material and methods

All the specimens examined in this study are deposited in the Institute of Entomology, College of Life Sciences, Nankai University, Tianjin, China (NKUM). All measurements are given in millimeters (mm). The illustrations of specimens in dorsal view and structural details were captured using a Nikon SMZ1000 stereomicroscope equipped with a computer-controlled SPOT RT digital camera and Helicon software (Helicon Remote ver. 3.9.12 W and Helicon Focus ver. 7.7.5). The skeletal elements of genital segments were dissected after being macerated with 5% KOH. The photographs of the dissected male genital segments immersed in glycerin were made using an OLYMPUS BX53 microscope equipped with a computer-controlled Canon OLYMPUS DP72 digital camera and cellSens Standard ver. 1.6 software. The recording points in the map refer to the distribution information from the literature (Tran et al. 2009; Jin et al. 2023).

## ﻿Taxonomic accounts

**Family Veliidae Brullé, 1836**

**Subfamily Veliinae Brullé, 1836**

**Genus *Velia* Latreille, 1804**

### Subgenus ﻿ Cesavelia

Taxon classificationAnimaliaHemipteraVeliidae

Koçak & Kemal, 2010

32E8B36A-284F-597C-B060-B458BED721A0

#### Diagnosis

(modified from Andersen 1981, and Tran et al. 2009). Antennal segment I distinctly longer than head width across eyes (except *Velia anderseni* Tran, Zettel & Buzzetti, 2009, which is almost equal to head width). Mesotarsus less than 2/3 mesotibia length. Metatibia distinctly longer than metafemur. Metafemur of male slender or moderately incrassate (in *V. anderseni*) and more heavily armed than in female on flexor side. Metatibia with scattered erect setae. In males, abdominal segment VIII relatively large, dorsal hind margin medially emarginated; proctiger plate-shaped, apically broadened; parameres prominent, strongly curved. In females, proctiger plate-shaped, covering gonocoxae and genital opening.

##### ﻿Key to the species of Velia (Cesavelia)

Note. Apterous forms; updated based on the “Key to the species of Velia (Haldwania) in the Oriental Region” in Tran et al. 2009). Hitherto, apterous forms of *V. championi* and *V. steelei* are still unknown (Tran et al. 2009).

**Table:** 

1	Tarsal segment II of hind leg subequal or shorter than segment III. Metafemur of male, width/length ratio ≈ 0.27 (fig. 5 in Tran et al. 2009)	***V. anderseni***
–	Tarsal segment II of hind leg distinctly longer than segment III (1.1–1.6 times). Metafemur of male, width/length ratio < 0.2 (Fig. 3)	**2**
2	Sides of sternites black and with wide yellow connexival stripes almost restricted to sternites III–V (macropterous morph; fig. 45 in Tran et al. 2009). Extensor sides of meso-and metatibiae with erect setae longer than tibia width (Tran et al. 2009)	***V. steelei***
–	Sides of sternites without such color pattern. Extensor sides of meso-and metatibiae with erect setae shorter than tibia width (Tran et al. 2009)	**3**
3	Sides of abdomen predominantly orange, black marks restricted to sternites II–IV (fig. 43 in Tran et al. 2009)	***V. championi***
–	Sides of abdomen predominantly black or dark brown (Tran et al. 2009)	**4**
4	Male	**5**
–	Female	**15**
5	Proctiger strongly modified, with large dilations on posterior part (figs 18–20 in Tran et al. 2009 and fig. 7a, b in Jin et al. 2023)	**6**
–	Proctiger only slightly modified or not modified, with small dilations on postero-lateral part (Fig. 4g, i, k; figs 14–16 in Tran et al. 2009; fig. 13 in Basu et al. 2013 and fig. 7c, d in Jin et al. 2023)	**9**
6	Hind margin of proctiger bisinuate, sides upcurved with pointed wings (fig. 18 in Tran et al. 2009)	***V. laticaudata***
–	Hind margin of proctiger not bisinuate (straight, nearly straight, or convex)	**7**
7	Hind margin of proctiger almost straight, lateral margin straight (fig. 20 in Tran et al. 2009)	***V. yunnana***
–	Hind margin of proctiger convex or curved, lateral margin curved	**8**
8	Hind margin of proctiger slightly curved (fig. 19 in Tran et al. 2009 and fig. 7b in Jin et al. 2023)	***V. longiconnexiva***
–	Hind margin of proctiger broadly rounded (fig. 7b in Jin et al. 2023)	***V. bui***
9	Apex of paramere moderately rounded (figs 25, 26 in Tran et al. 2009 and figs 15, 16 in Basu et al. 2013). Endosoma: accessory sclerite well-sclerotized, ventral sclerites visible (fig. 37 in Tran et al. 2009 and fig. 14 in Basu et al. 2013)	**10**
–	Apex of paramere rather acute. Endosma: accessory sclerite and ventral sclerites not visible	**11**
10	Metafemur with two long, divergent teeth on basal half (figs 11–12 in Basu et al. 2013)	***V. mitrai***
–	Metafemur with one long tooth on basal half (fig. 3 in Tran et al. 2009)	***V. tomokunii***
11	Sub-apical part of paramere with distinct constriction (Fig. 4n). Endosma: lateral sclerites large, strongly sclerotized, dorsal sclerites distinctly sclerotized, medially with membranous parts (Fig. 4r, s)	***V. motuoensis* sp. nov.**
–	Sub-apical part of paramere without distinct constriction. Endosma: lateral sclerites slender, dorsal sclerites weakly sclerotized (Fig. 4p, q, t, u)	**12**
12	Body distinctly brighter, with large, bright yellow stripes along entire connexiva, median parts of mediotergites with distinct dark orange marks (Fig. 2k–l)	***V. yiliangensis* sp. nov.**
–	Body darker, with relative narrow orange stripes along connexiva, median parts of mediotergites without distinct dark orange marks (fig. 2d, f in Jin et al. 2023)	**13**
13	Stripes along almost the entire connexiva except the posterior part of connexivum VII distinctly brighter (Fig. 2g, h). Prominent sub-apical tooth on the ventral side of the male metafemur significantly larger than the sub-basal one (Fig. 3a)	***V. lii* sp. nov.**
–	Stripes along the connexiva relatively dull or posteriorly reduced (fig. 2d, f in Jin et al. 2023). Prominent sub-apical tooth on the ventral side of the male metafemur not distinctly larger than the sub-basal one (fig. 5c, d in Jin et al. 2023 and figs 2, 4 in Tran et al. 2009)	**14**
14	Antennal segment I at least 1.6 times head width	***V. sinensis***
–	Antennal segment I around 1.4 times head width	***V. tonkina***
15	Proctiger ovate, longer than broad (fig. 70 in Tran et al. 2009). Tergite VIII only with very short pubescence on hind margin. Connexiva swollen on segments III–IV; connexival spines long and caudally directed (figs 56– 57 in Tran et al. 2009)	***V. laticaudata***
–	Proctiger about as long as broad or distinctly broader than long. Tergite VIII with more or less long hairs on hind margin. Connexiva not swollen; connexival spines short or dorso-caudally directed	**16**
16	Proctiger with more or less angulated sides (Fig. 4h, j, l; fig. 64–68 in Tran et al. 2009; fig. 17 in Basu et al. 2013 and fig. 7g–d in Jin et al. 2023)	**17**
–	Proctiger with rounded sides (fig. 71, 72 in Tran et al. 2009 and fig. 7e, f in Jin et al. 2023)	**22**
17	Proctiger much broader than long (Fig. 4j). Lateral parts of mediotergite I and laterotergites covered with dense silvery pubescence (Fig. 2c)	***V. motuoensis* sp. nov.**
–	Proctiger slightly broader than long. Lateral parts of mediotergite I and laterotergites covered with sparse silvery pubescence	**18**
18	Stripes along entire connexiva wide and bright yellow, median parts of mediotergites with distinct dark orange marks (Fig. 2e, f)	***V. yiliangensis* sp. nov.**
–	Without such remarkable stripes along connexiva, median parts of mediotergites without distinct dark orange marks	**19**
19	Narrow stripes along almost the entire connexiva, except the posterior part of connexivum VII distinctly brighter (Fig. 2a, b)	***V. lii* sp. nov.**
–	Narrow stripes along connexiva dull or posteriorly reduced (fig. 3e–h in Jin et al. 2023 and figs 4, 6 in Basu et al. 2013)	**20**
20	Ventral part of abdomen bright orange (figs 4, 6, 10 in Basu et al. 2013)	***V. mitrai***
–	Ventral part of abdomen dull or black (figs 3f, h, 4b, d, f, h, j, l in Jin et al. 2023)	**21**
21	Antennal segment I at least 1.6 times head width	***V. sinensis***
–	Antennal segment I about 1.3 times head width	***V. tonkina***
22	Proctiger with maximum width close to base, the basal part of lateral sides more convex than distal part (fig. 72 in Tran et al. 2009)	***V. yunnana***
–	Proctiger with maximum width approximately at mid-length	**23**
23	Proctiger with sides broadly rounded (fig. 7e, f in Jin et al. 2023). Connexival spines long (length of connexival spines distinctly greater than maximum width) and dorso-caudally directed (figs 58, 59 in Tran et al. 2009 and fig. 3a–d in Jin et al. 2023)	**24**
–	Proctiger with sides narrower (figs 65, 66 in Tran et al. 2009). Connexival spines short (length of connexival spines shorter than maximum width, or spines instinct) and caudally directed (figs 49–51 in Tran et al. 2009)	***V. tomokunii***
24	Connexiva nearly straight and almost parallel, connexival spines gradually sharpened in lateral view (Fig. 3a, b)	***V. bui***
–	Connexiva convergent, connexival spines not gradually sharpened, sub-apical part slightly narrower than basal part in lateral view (fig. 3c, d in Jin et al. 2023 and figs 58, 59 in Tran et al. 2009)	***V. longiconnexiva***

### ﻿ Velia (Cesavelia) lii sp. nov.

Taxon classificationAnimaliaHemipteraVeliidae

6F5ABD44-A6B1-5791-BF1B-EE9A0AFA84A6

https://zoobank.org/3C57DBF6-F6E0-442F-90B7-26F1CA99E68E

**Figure 1.**
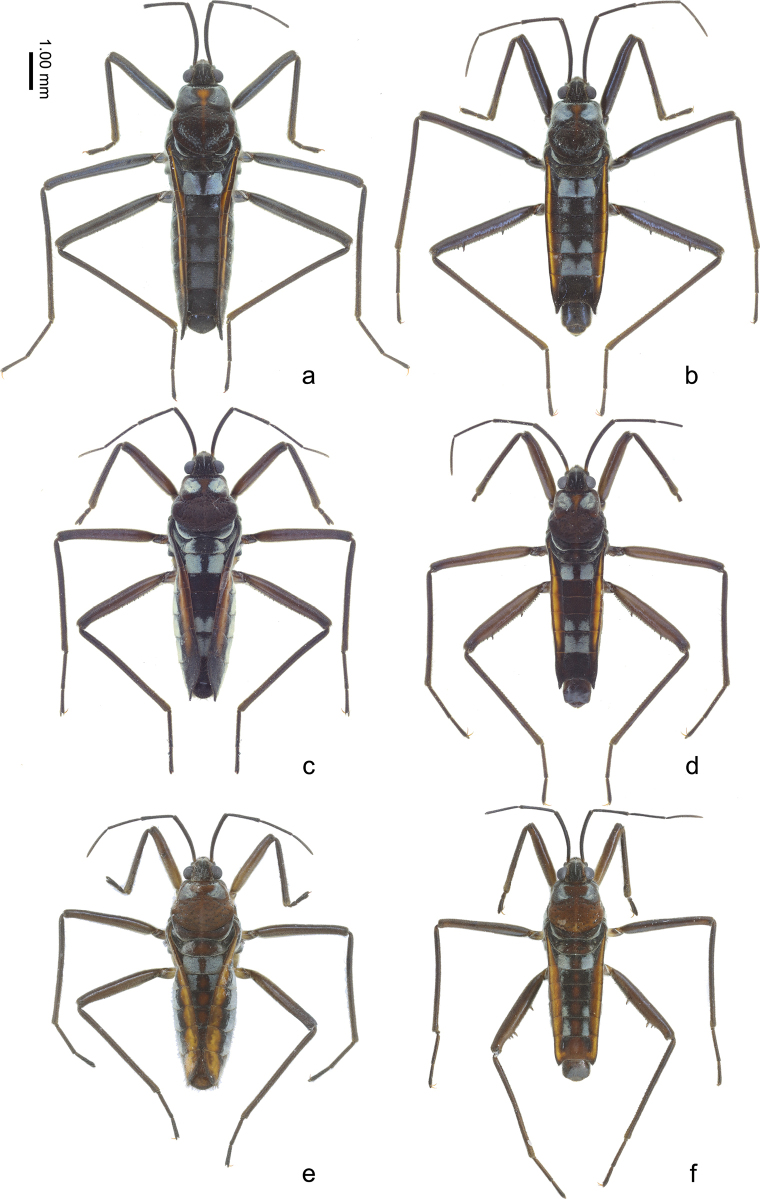
Habitus of females and males of Velia (Cesavelia) lii sp. nov. (a, b), Velia (Cesavelia) motuoensis sp. nov. (c, d) and Velia (Cesavelia) yiliangensis sp. nov. (e, f) in dorsal view. a, c, e apterous female b, d, f apterous male.

**Figure 2.**
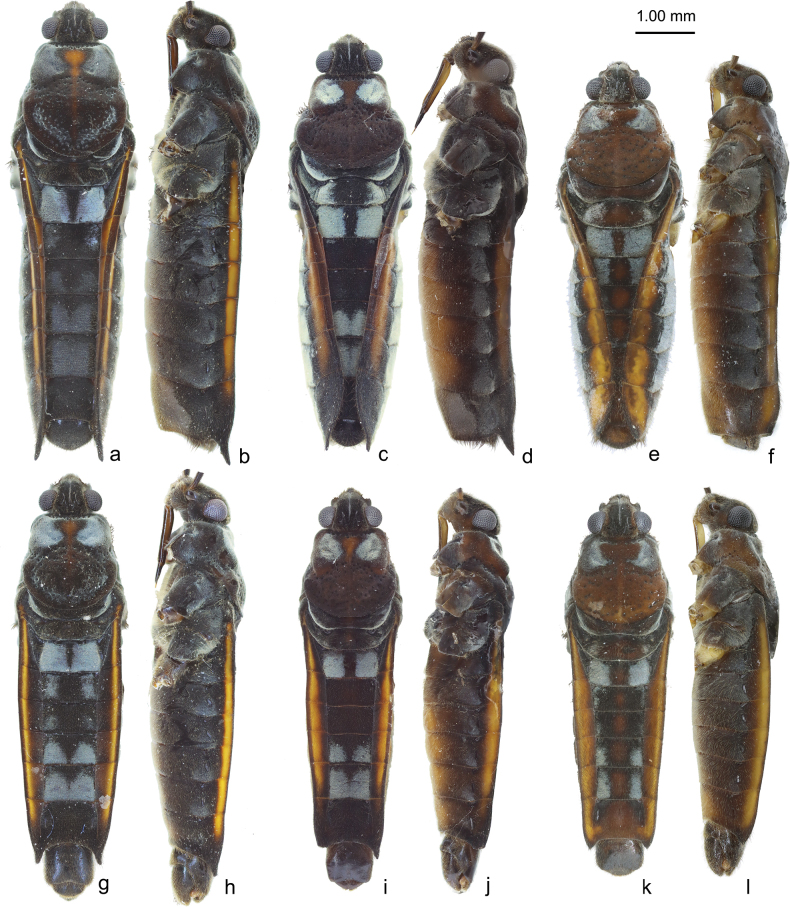
Bodies of *V. lii* sp. nov. (a, b, g, h), *V. motuoensis* sp. nov. (c, d, i, j) and *V. yiliangensis* sp. nov. (e, f, k, l). a–f. Apterous female; g–l. Apterous male.

**Figure 3.**
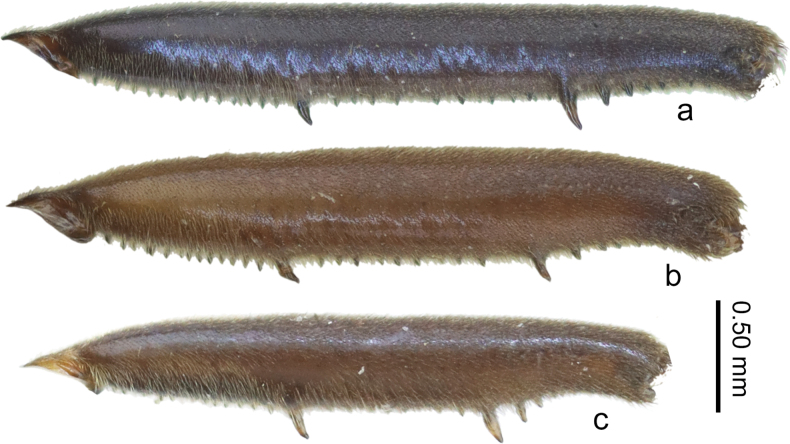
Metafemora of males, showing patterns of spines. a. *V. lii* sp. nov.; b. *V. motuoensis* sp. nov.; c. *V. yiliangensis* sp. nov.

**Figure 4.**
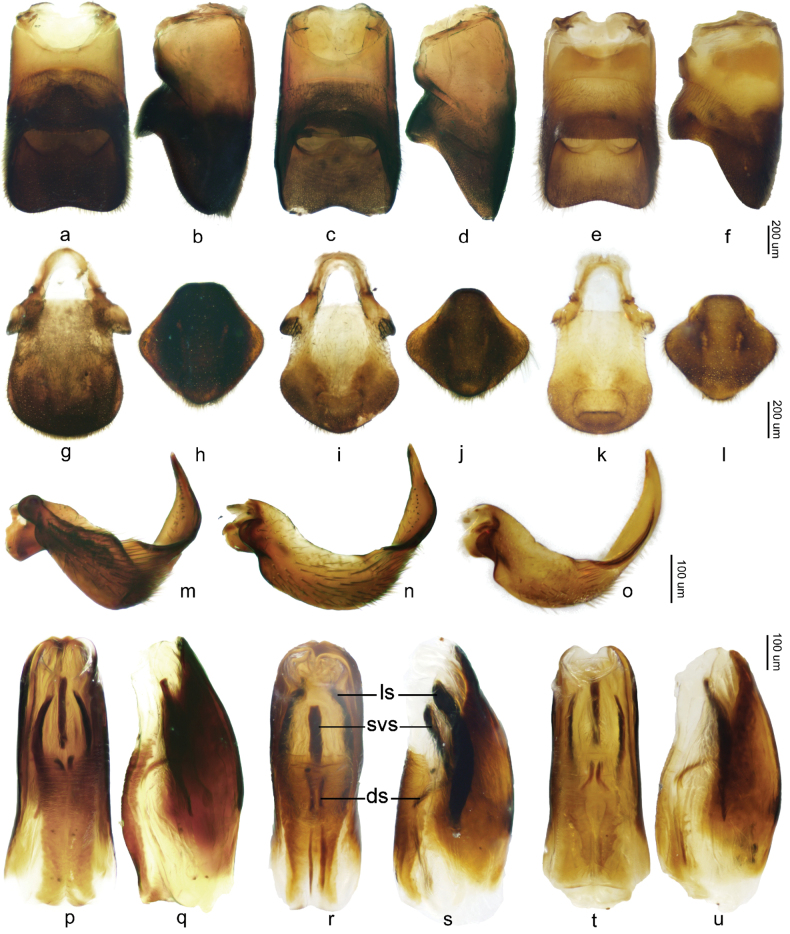
Abdominal segments VIII of males and genital structures of *Velia* spp., a–f. Abdominal segments VIII of males (a, c, e in ventral view b, d, f in lateral view); g, i, k. proctiger of male; h, j, l. Proctiger of female; m–o. Left paramere of males; p–u. Endosomal structure of males (p, r, t in dorsal view q, s, u in lateral view). a–b, g–h, m, p–q. *V. lii* sp. nov.; c–d, i–j, n, r–s. *V. motuoensis* sp. nov.; e–f, k–l, o, t–u. *V. yiliangensis* sp. nov. (ds = dorsal sclerite, ls = lateral sclerite, svs = secondary ventral sclerite).

Figs 1a, b, 2a, b, g, h, 3a, 4a, b, g, h, m, p, q

#### Material examined.

***Holotype***: apterous ♂, China • Xizang Province, Linzhi City, Motuo County, Bangxin Village: 29.5763°N, 95.4643°E; 1360 m a.s.l.; 2024-VIII-19; Zihe Li leg. (NKUM). ***Paratypes***: 1 apterous ♀, same data as holotype (NKUM).

#### Diagnosis.

Body large (length 7.73–7.90, width 2.00–2.50), mainly brown. Connexiva of apterous female slightly curved in dorsal view, with bright yellow stripes along almost entire connexiva except posterior part of connexivum VII in both sexes (Figs 1a, b, 2a, b, g, h), connexival spines sharp and caudally directed in male, dorso-caudally directed in female (Fig. 2a, b, g, h); abdominal segment VIII of male stout and ventrally concave (Fig. 4a, b); proctiger of male broad, shield-shaped, posterior margin rounded (Fig. 4g); paramere broad, strongly curved, with thick setae on external side, apices sharp (Fig. 4m); endosoma of male stout, apical ends of lateral sclerites distinctly constricted, dorsal sclerites weakly sclerotized, translucent and curved, secondary ventral sclerite slender, accessory sclerite absent (Fig. 4p, q); proctiger of female broad, diamond-shaped (Fig. 4h).

#### Comparative notes.

Velia (Cesavelia) lii sp. nov. is most similar to *V. bui* in appearance. It can be distinguished from other species of *Cesavelia* by the following characters: the absence of the accessory sclerite in the male endosoma distinguishes this species from *V. tomokunii* J. Polhemus & D. Polhemus, 2003, *V. championi* Tamanini, 1955, and *V. mitrai* Basu, Subramanian & D. Polhemus, 2013 (Fig. 4p, q); the shape of the male proctiger (Fig. 4g) distinguishes this species from *V. bui* Jin, Fu & Ye, 2023, *V. longiconnexiva* Tran, Zettel & Buzzetti, 2009, *V. anderseni* Tran, Zettel & Buzzetti, 2009, *V. laticaudata* Tran, Zettel & Buzzetti, 2009, and *V. yunnana* Tran, Zettel & Buzzetti, 2009; and the bright yellow stripes along almost the entire connexiva except the posterior part of connexivum VII in both sexes (Figs 1a, b, 2a, b, g, h), and the prominent sub-apical tooth on the ventral side of the male metafemur, which is significantly larger than the sub-basal one (Fig. 3a), distinguish this species from *V. sinensis* and *V. tonkina* D. Polhemus & J. Polhemus, 2003. Due to the fact that *V. steelei* Tamanini, 1955 is only known from the macropterous female, and only the apterous female and male of *V. lii* sp. nov. have been collected to date, a comparison between the same forms is impossible (Tamanini 1995a). However, we note that *V. steelei* possesses long, erect setae on the extensor sides of the meso- and metatibia, these being longer than the tibial width (Tran et al. 2009). This characteristic is considered diagnostic (Tran et al. 2009) and is not found in *V. lii* sp. nov., thereby allowing for the distinction between these two species. Furthermore, the differences of the dorsal sclerites and lateral sclerites of the male endosoma distinguish *V. lii* sp. nov. from Velia (Cesavelia) motuoensis sp. nov. (Fig. 4p, q vs. Fig. 4r, s); and the larger body size and the differences of yellow stripes along the connexiva distinguish it from Velia (Cesavelia) yiliangensis sp. nov. (Fig. 2a, b, g, h vs. Fig. 2e, f, k, l).

#### Description.

**Apterous male (holotype). *Measurements*.** Body: length 7.73, width 2.00. Head: length 0.91, width: 1.16. Antenna: 5.75(1.98+1.26+1.23+1.28), length of antennal segment I about 1.71 times head width. Pronotum: width about 0.98 times its length (length 1.68, width 1.65). Lengths of leg segments (femur: tibia: tarsus (tarsal segment I + segment II + segment III)): fore leg: 2.47: 2.47: 0.96 (0.13+0.27+0.56); middle leg: 3.40: 3.90: 2.21 (0.10+1.20+0.91), length of mesotarsus II about 1.32 times length of mesotarsus III; hind leg: 3.40: 4.25: 2.07 (0.10+1.13+0.84), maximum width of metafemur: 0.42, length of metatarsus II about 1.35 times length of metatarsus III.

***Color*** (Figs 1b, 2g, h). Body mainly dark brown, with scattered silvery pubescence. Pronotum with a row of black punctures near anterior margin and other punctures scattered on posterior lobe. Median part of anterior pronotal lobe and midline of pronotum dark orange. Sides of abdomen dark brown, with bright orange stripes along almost entire connexiva except posterior part of connexivum VII. Silvery pubescence usually distinctly denser on anterolateral corners of pronotum, lateral corners of metanotum, lateral parts of abdominal mediotergites II, V–VI, sparse on abdominal mediotergites I, III–IV and lateral parts of sternites.

***Structure*.** Body relatively large, covered with dense, short pubescence. ***Head*** (Figs 1b, 2g, h): triangular, almost perpendicular to thorax, without deflection; anteclypeus and postclypeus with dense, peg-like setae; antennal sockets prominent, antennal segment I much longer than head width, slightly thicker than antennal segments II–IV. ***Thorax*** (Figs 1b, 2g, h): width and length of pronotum approximately equal, posterior margin of pronotum broadly rounded, lateral parts of pronotum distinctly constricted at mid-length, median part slightly raised and lateral parts of anterior pronotal lobe concave; mesonotum completely hidden beneath pronotal lobe, with hind part of metanotum visible in dorsal view; lateral evaporatoriums slender, with a cluster of suberect, thick setae on each side; legs mainly with decumbent or suberect setae, tarsi of fore legs short, tarsi of middle and hind legs long and slender; profemora moderately incrassate, slightly curved and contracted subapically; mesofemora slender; metafemora (Fig. 3a) relatively slender, ventrally with two rows of small teeth and two prominent long teeth, the sub-apical tooth significantly larger and more prominent than the sub-basal one, metatibiae ventrally with two rows of small spines. ***Abdomen*** (Figs 1b, 2g, h): relatively slender; mediotergite I concave laterally, mediotergites II–VII almost flat; connexiva moderately raised, almost parallel, hardly converging, connexival spines short, sharp, caudally pointed; abdominal segment VIII (Fig. 4a, b) relatively stout, ventrally concave in lateral view, posteriorly with short, dense setae, posterodorsal margin of abdominal segment VIII medially emarginated. ***Genital segments*** (Fig. 4g, m, p, q): relatively large and visible in vitro; proctiger (Fig. 4g) shield-shaped, posterior margin rounded, with short, sparse setae; paramere (Fig. 4m) broad, strongly curved, with thick setae on external side, apices sharp; endosoma (Fig. 4p, q) stout, apical ends of lateral sclerites distinctly constricted, dorsal sclerites weakly sclerotized, translucent and curved, secondary ventral sclerite slender, accessory sclerite absent.

**Apterous female. *Measurements*.** Body: length 7.90, width 2.50. Head: length 0.96, width: 1.24. Antenna I–III: (1.87+1.22+1.27), length of antennal segment I about 1.51 times head width. Pronotum: width about 0.94 times length (length 2.00, width 1.88). Lengths of leg segments (femur: tibia: tarsus (tarsal segment I + segment II + segment III)): fore leg: 2.70: 2.53: 0.98 (0.09+0.31+0.58); middle leg: 3.65: 4.00: 2.28 (0.11+1.24+0.93), length of mesotarsus II about 1.33 times length of mesotarsus III; hind leg: 3.65: 4.20: 2.21 (0.16+1.18+ 0.87), length of metatarsus II about 1.36 times length of metatarsus III.

***Color*** (Figs 1a, 2a, b). Similar to apterous male with following exceptions: silvery pubescence weaker on abdominal mediotergites III–VI (Fig. 2a).

***Structure*.** Body slightly larger than apterous male. ***Head*** (Figs 1a, 2a, b): Similar to apterous male. ***Thorax*** (Figs 1a, 2a, b): similar to apterous male with following exceptions: profemora slender; metafemora slender, ventrally with two rows of small spines, metatibiae ventrally without any spines or teeth. ***Abdomen*** (Figs 1a, 2a, b): similar to apterous male with following exceptions: relatively stout; connexiva slightly convergent towards abdominal apex, connexival spines long, slender and straight, dorso-caudally directed. ***Genital segments***: gonocoxae and gonapophyses semi-membranous, rami strongly sclerotized; proctiger (Fig. 4h) broad, diamond-shaped, posteriorly with short, sparse setae.

#### Macropterous female and macropterous male.

Unknown.

#### Etymology.

The species is named in honor of Dr Zihe Li, who is the only one to successfully collect the specimens from a hazardous mountain stream.

#### Habitats.

**Figure 5.**
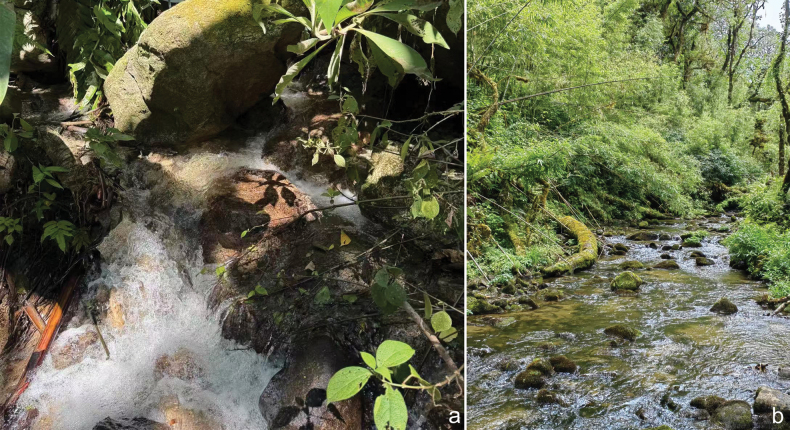
Photographs of habitats of *Velia* spp., a. Habitat of *V. lii* sp. nov.; b. Habitat of *V. motuoensis* sp. nov.

This species inhabits areas near rocks or the banks of fast-flowing mountain streams (Fig. 5a).

#### Distribution.

**Figure 6.**
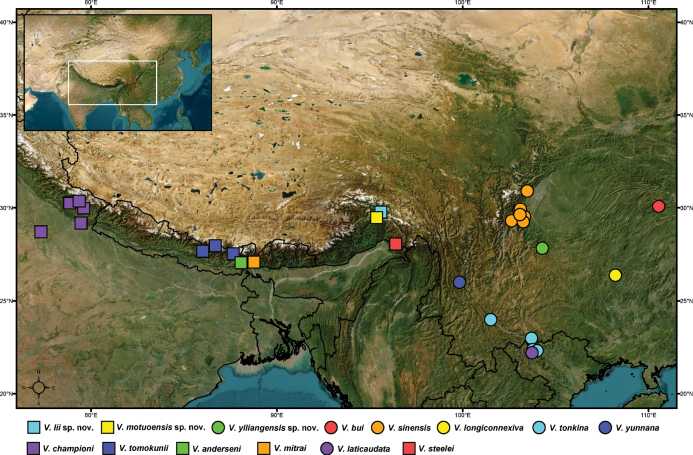
Geographical distribution of the subgenus Velia (Cesavelia) to date.

China (Xizang) (Fig. 6).

### ﻿ Velia (Cesavelia) motuoensis sp. nov.

Taxon classificationAnimaliaHemipteraVeliidae

F9C99836-0699-58A7-B9CB-AB04D2CAA3EE

https://zoobank.org/7E0F62B1-16AD-459B-8A0C-C5EC0AF50E46

Figs 1c, d, 2c, d, i, j, 3b, 4c, d, i, j, n, r, s

#### Material examined.

***Holotype***: apterous ♂, China • Xizang Province, Linzhi City, Motuo County, Motuo Village: 29.3051°N, 95.3567°E; 1936 m a.s.l.; 2024-VIII-20; Zezhong Jin, Zihe Li leg. (NKUM). ***Paratypes***: 1 apterous ♂, 3 apterous ♀, same data as holotype (NKUM).

#### Diagnosis.

Body large (length 6.90–7.10, width 2.00–2.25), mainly brown. Connexiva of apterous female slightly curved, convergent posteriorly in dorsal view, with orange stripes along connexiva in both sexes (Figs 1c, d, 2c, d, i, j), connexival spines sharp and caudally directed in males, dorso-caudally directed in females (Fig. 2c, d, i, j); abdominal segment VIII of male stout and ventrally concave (Fig. 4c, d); proctiger of male broad, shield-shaped, posterior margin rounded (Fig. 4i); paramere broad, strongly curved, with thick setae on external side, apices sharp (Fig. 4n); endosoma of male stout, lateral sclerites large, strongly sclerotized, apical ends of lateral sclerites distinctly constricted, dorsal sclerites distinctly sclerotized, translucent and curved, with membranous parts medially, secondary ventral sclerite stout, accessory sclerite absent (Fig. 4r, s); proctiger of female broad, diamond-shaped, lateral dilations distinct (Fig. 4j).

#### Comparative notes.

Velia (Cesavelia) motuoensis sp. nov. is most similar to *V. longiconnexiva* in appearance. It can be distinguished from other species of *Cesavelia* by the following characters: the absence of the accessory sclerite in the male endosoma distinguishes this species from *V. tomokunii*, *V. championi*, and *V. mitrai* (Fig. 4r, s); the shape of the male proctiger (Fig. 4i) distinguishes this species from *V. bui*, *V. longiconnexiva*, *V. anderseni*, *V. laticaudata*, and *V. yunnana*; and the characteristics of the endosomal sclerites, especially the large lateral sclerites and dorsal sclerites with membranous parts medially, distinguish this species from *V. sinensis*, *V. tonkina*, *V. lii* sp. nov. and *V. yiliangensis* sp. nov. (Fig. 4r, s vs. Fig. 4p, q, t, u). The extensor sides of the meso- and metatibia without long, erect setae and the shape of the female proctiger (Fig. 4j) distinguish this species from *V. steelei*.

#### Description.

**Apterous male (holotype). *Measurements*.** Body: length 6.90 (6.90–7.10), width 2.00 (2.00–2.13). Head: length 0.70, width: 1.15. Antenna: 5.43 (1.89+1.27+1.20+1.07), length of antennal segment I about 1.64 times head width. Pronotum: width about 1.12 times its length (length 1.50, width 1.68). Lengths of leg segments (femur: tibia: tarsus (tarsal segment I + segment II segment III)): fore leg: 2.50: 2.27: (0.09+0.24+(segment III missing)); middle leg: 3.30: 3.50: 2.13 (0.13+1.16+ 0.84), length of mesotarsus II about 1.38 times length of mesotarsus III; hind leg: 3.25: 3.55: 1.99 (0.12+1.07+ 0.80), maximum width of metafemur: 0.51, length of metatarsus II about 1.34 times length of metatarsus III.

***Color*** (Figs 1d, 2i, j). Body mainly dark brown, with scattered silvery pubescence. Pronotum with a row of black punctures near anterior margin and other punctures scattered on posterior lobe. Median part of anterior pronotal lobe dark orange. Sides of abdomen dark brown, with bright orange stripes along connexival segments II–VI, the width of stripes exhibits intraspecific variations; in some samples, the stripes almost cover the entire connexiva. Silvery pubescence usually distinctly denser on anterolateral corners of pronotum, lateral corners of metanotum, lateral parts of abdominal mediotergites II, V–VI, sparse on abdominal mediotergite I and lateral parts of sternites.

***Structure*.** Body relatively large, covered with dense, short pubescence. ***Head*** (Figs 1d, 2i, j): triangular, almost perpendicular to thorax, without deflection; anteclypeus and postclypeus with dense, peg-like setae; antennal sockets prominent, antennal segment I much longer than head width, slightly thicker than antennal segments II–IV. ***Thorax*** (Figs 1d, 2i, j): pronotum slightly wider than length, posterior margin of pronotum broadly rounded, lateral parts of pronotum distinctly constricted at mid-length, middle part slightly raised and lateral parts of anterior pronotal lobe concave; mesonotum completely hidden beneath pronotal lobe and hind part of metanotum visible in dorsal view; lateral evaporatoriums slender, with a cluster of suberect, thick setae on each side; legs mainly with decumbent or suberect setae, tarsi of fore legs short, tarsi of middle and hind legs long and slender; profemora moderately incrassate, slightly curved and contracted subapically; mesofemora slender; metafemora (Fig. 3b) relatively slender, ventrally with two rows of small teeth and two prominent long teeth, the sub-apical tooth almost as long as the sub-basal one, metatibiae ventrally with two rows of small spines. ***Abdomen*** (Figs 1d, 2i, j): relatively slender; mediotergite I concave laterally, mediotergites II–VII almost flat; connexiva moderately raised, almost parallel, hardly converging, connexival spines short, sharp, caudally pointed; abdominal segment VIII (Fig. 4c, d) relatively stout, ventrally concave in lateral view, posteriorly with short, sparse setae, posterodorsal margin of abdominal segment VIII medially emarginate. ***Genital segments*** (Fig. 4i, n, r, s): relatively large and visible in vitro; proctiger (Fig. 4i) shield-shaped, hind margin rounded, posterior with short, sparse setae; paramere (Fig. 4n) broad, strongly curved, with thick setae on external side, apices sharp; endosoma (Fig. 4r, s) stout, lateral sclerites large, strongly sclerotized, apical ends of lateral sclerites distinctly constricted, dorsal sclerites distinctly sclerotized, translucent and curved, medially with membranous parts, secondary ventral sclerite stout, accessory sclerite absent.

**Apterous female. *Measurements*.** Body: length 7.00–7.10, width 2.20–2.25. Head: length 0.82, width: 1.16. Antenna: 4.97 (1.71+1.11+1.07+1.08), length of antennal segment I about 1.47 times head width. Pronotum: width about 1.09 times its length (length 1.68, width 1.83). Lengths of leg segments (femur: tibia: tarsus (tarsal segment I + segment II + segment III)): fore leg: 2.35: 2.05: 0.82 (0.09+0.24+0.49); middle leg: 3.17: 3.40: 2.02 (0.11+1.11+ 0.80), length of mesotarsus II about 1.39 times length of mesotarsus III; hind leg: 3.15: 3.55: 1.94 (0.13+1.04+ 0.77), length of metatarsus II about 1.35 times length of metatarsus III.

***Color*** (Figs 1a, 2a, b). Similar to apterous male with following exceptions: silvery pubescence denser on lateral parts of abdominal mediotergite I and laterotergites I (Fig. 2c). The width of stripes on connexiva exhibits intraspecific variations, wider and brighter in one sample.

***Structure*.** Body slightly larger than apterous male. ***Head*** (Figs 1c, 2c, d): Similar to apterous male. ***Thorax*** (Figs 1c, 2c, d): similar to apterous male with following exceptions: profemora slender; metafemora slender, ventrally with two rows of small spines, metatibiae ventrally with some sparse small spines. ***Abdomen*** (Figs 1c, 2c, d): similar to apterous male with following exceptions: relatively stout; connexiva gradually convergent towards abdominal apex, connexival spines long, slender and straight, dorso-caudally directed. ***Genital segments***: gonocoxae and gonapophyses semi-membranous, rami strongly sclerotized; proctiger (Fig. 4j) broad, diamond-shaped, lateral dilations distinct, posteriorly with short, sparse setae.

#### Macropterous female and macropterous male.

Unknown.

#### Etymology.

This species is named after its type locality, Motuo Village, Motuo County, China.

#### Habitats.

This species is found in the nearshore areas of larger, slow-flowing streams, particularly in corners formed by deadwood or vegetation. (Fig. 5b).

#### Distribution.

China (Xizang) (Fig. 6).

### ﻿ Velia (Cesavelia) yiliangensis sp. nov.

Taxon classificationAnimaliaHemipteraVeliidae

8E26DFF7-CE6A-5D62-97ED-CA1F2BBE1EDD

https://zoobank.org/3D74F8D4-7E18-4611-99B7-36A5BE603BAC

Figs 1e, f, 2e, f, k, l, 3c, 4e, f, k, l, o, t, u

#### Material examined.

***Holotype***: apterous ♂, China • Yunnan Province, Zhaotong City, Yiliang County, Xiaocaoba Scenic Area: 27.8271°N, 104.2869°E; 1758 m a.s.l.; 2020-VIII-19; Mu Qiao leg. (NKUM). ***Paratypes***: 1 apterous ♂, 5 apterous ♀, same data as holotype (NKUM).

#### Diagnosis.

Body medium (length 6.33–6.80, width 1.82–2.27), mainly medium brown. Connexiva of apterous female curved, convergent in dorsal view posteriorly, with large, bright yellow stripes along entire connexiva in both sexes (Figs 1e, f, 2e, f, k, l), connexival spines short, caudally directed in males, absent in females (Fig. 2e, f, k, l); abdominal segment VIII of male stout and ventrally concave (Fig. 4e, f); proctiger of male broad, shield-shaped, posterior margin rounded (Fig. 4k); paramere relatively slender, strongly curved, with thick setae on external side, apices sharp (Fig. 4o); endosoma of male stout, apical ends of lateral sclerites constricted, dorsal sclerites distinctly weakly sclerotized, translucent and curved, secondary ventral sclerite slender, accessory sclerite absent (Fig. 4t,u); proctiger of female broad, diamond-shaped (Fig. 4l).

#### Comparative notes.

Velia (Cesavelia) yiliangensis sp. nov. is most similar to *V. sinensis* in appearance. It can be distinguished from other species of *Cesavelia* by the following characters: the absence of the accessory sclerite in the male endosoma distinguishes this species from *V. tomokunii*, *V. championi*, and *V. mitrai* (Fig. 4t, u); the shape of the male proctiger (Fig. 4k) distinguishes this species from *V. bui*, *V. longiconnexiva*, *V. anderseni*, *V. laticaudata*, and *V. yunnana*; and the entire connexiva with large, bright yellow stripes in both sexes, and two less prominent teeth near the sub-apical prominent tooth on the ventral side of the male metafemur (Figs 1f, 3c), distinguish this species from *V. sinensis* and *V. tonkina*. The extensor sides of the meso- and metatibia without long, erect setae distinguish this species from *V. steelei*. The large, bright yellow stripes along the entire connexiva in both sexes distinguish it from other new species in this study (Fig. 2e, f, k, l vs. Fig. 2a–d, 2g–j).

#### Description.

**Apterous male (holotype). *Measurements*.** Body: length 6.47 (6.43–6.47), width 1.82 (1.82–2.02). Head: length 0.58, width: 1.11. Antenna: 4.59 (1.49+1.00+ 1.01+ 1.09), length of antennal segment I about 1.34 times head width. Pronotum: width about 1.14 times its length (length 1.40, width 1.60). Lengths of leg segments (femur: tibia: tarsus (tarsal segment I + segment II + segment III)): fore leg: 2.07: 2.03: 0.74 (0.07+0.20+0.47); middle leg: 2.90: 3.15: 1.85 (0.09+1.00+0.76), length of mesotarsus II about 1.32 times length of mesotarsus III; hind leg: 2.80: 3.30: 1.67 (0.09+ 0.89+ 0.69), maximum width of metafemur: 0.42, length of metatarsus II about 1.29 times length of metatarsus III.

***Color*** (Figs 1f, 2k, l). Body mainly brown, with scattered silvery pubescence. Pronotum with a row of black punctures near anterior margin and other punctures scattered on posterior lobe. Median part of posterior pronotal lobe pale brown. Sides of abdomen dark brown, with large, bright yellow stripes along entire connexiva; median parts of mediotergites with some dark orange marks, in another male brighter. Silvery pubescence usually distinctly denser on anterolateral corners of pronotum, lateral corners of metanotum, lateral parts of abdominal mediotergites II, V–VI, sparse on abdominal mediotergites I, III–IV and sternites.

***Structure*.** Body relatively large, covered with dense, short pubescence. ***Head*** (Figs 1f, 2k, l): triangular, almost perpendicular to thorax, without deflection; anteclypeus and postclypeus with dense, peg-like setae; antennal sockets prominent, antennal segment I much longer than head width, slightly thicker than antennal segments II–IV. ***Thorax*** (Figs 1f, 2k, l): pronotum slightly wider than length, posterior margin of pronotum broadly rounded, lateral parts of pronotum distinctly constricted at mid-length,, middle part slightly raised and lateral parts of anterior pronotal lobe concave; mesonotum completely hidden beneath pronotal lobe and hind part of metanotum visible in dorsal view; lateral evaporatoriums slender, with a cluster of suberect, thick setae on each side; legs mainly with decumbent or suberect setae, tarsi of fore legs short, tarsi of middle and hind legs long and slender; profemora moderately incrassate, slightly curved and contracted subapically; mesofemora medially slender; metafemora (Figs 1f, 3c) relatively slender, ventrally with two rows of small teeth and two prominent long teeth, the sub-apical tooth stouter than the sub-basal one, two long less prominent small teeth present near the sub-apical prominent tooth, are long, metatibiae ventrally with two rows of small spines. ***Abdomen*** (Figs 1f, 2k, l): relatively slender; mediotergite I concave laterally, mediotergites II–VII almost flat; connexiva moderately raised, almost parallel hardly converging, connexival spines very short, sharp, caudally pointed; abdominal segment VIII (Fig. 4e, f) relatively stout, ventrally concave in lateral view, posteriorly with short, dense setae, posterodorsal margin medially emarginate. ***Genital segments*** (Fig. 4k, o, t, u): relatively large and visible in vitro; proctiger (Fig. 4k) shield-shaped, posterior margin rounded, posteriorly with short, sparse setae; paramere (Fig. 4o) relatively slender, strongly curve, with thick setae on external side, apices sharp; endosoma (Fig. 4t, u) stout, apical ends of lateral sclerites slightly constricted, dorsal sclerites weakly sclerotized, translucent and curved, secondary ventral sclerite slender, accessory sclerite absent.

**Apterous female. *Measurements*.** Body: length 6.33–6.80, width 2.13–2.27. Head: length 0.62, width: 1.07. Antenna: 4.26 (1.40+0.93+0.93+1.00), length of antennal segment I about 1.31 times head width. Pronotum: width about 1.34 times its length (length 1.33, width 1.78). Lengths of leg segments (femur: tibia: tarsus (tarsal segment I + segment II + segment III)): fore leg: 2.10: 1.95: 0.72 (0.09+0.16+0.47); middle leg: 2.83: 3.17: 1.73 (0.09+0.91+0.73), length of mesotarsus II about 1.25 times length of mesotarsus III; hind leg: 2.70: 3.30: 1.52 (0.09+0.80+ 0.63), length of metatarsus II about 1.27 times length of metatarsus III.

***Color*** (Figs 1e, 2e, f). Similar to apterous male with following exception: in some females, bright yellow stripes along connexiva much wider than in male (Fig. 2e). Mediotergites of some females exhibit brighter yellow coloration. Silvery pubescence denser on lateral parts of mediotergites I and III (Fig. 2e).

***Structure*.** Body slightly larger than apterous male. ***Head*** (Figs 1e, 2e, f): Similar to apterous male. ***Thorax*** (Figs 1e, 2e, f): similar to apterous male with following exceptions: profemora much slender; metafemora more slender, ventrally with two rows of small spines on each side, metatibiae ventrally without any spines or teeth. ***Abdomen*** (Figs 1e, 2e, f): similar to apterous male with following exceptions: relatively stout; connexiva curved, gradually convergent towards abdominal apex, connexival spines absent. ***Genital segments***: gonocoxae and gonapophyses semi-membranous, rami strongly sclerotized; proctiger (Fig. 4l) broad, diamond-shaped, posteriorly with short, sparse setae.

#### Macropterous female and macropterous male.

Unknown.

#### Etymology.

This species is named after its type locality, Yiliang County, Yunnan Province, China.

#### Distribution.

China (Yunnan) (Fig. 6).

## ﻿Discussion

The sclerites of the endosoma in males of the subgenus Cesavelia had been previously discussed by Tran et al. (2009). While it was once believed that the sclerites exhibited little diversification within *Cesavelia*, our results reveal that the sclerites demonstrate a degree of variation. Some features appear to provide significant evidence of phylogenetic relationships. For example, accessory sclerites are only observable in *V. tomokunii*, *V. championi*, and *V. mitrai* (Tran et al. 2009), which suggests that these three species may share a closer phylogenetic relationship.

Furthermore, regarding the male proctiger, *V. bui*, *V. longiconnexiva*, *V. laticaudata*, and *V. yunnana* exhibit distinct lateral lobe-shaped dilations (Tran et al. 2009; Jin et al. 2023), while the remaining Velia (Cesavelia) species share more similar morphological shapes. The species exhibiting these similar characteristics also demonstrate distinct patterns in their distribution. *Velia tomokunii*, *V. championi*, and *V. mitrai* are found in the western regions of the southern slopes of the Himalayas (Fig. 6), whereas *V. bui*, *V. longiconnexiva*, *V. laticaudata*, and *V. yunnana* are distributed across the Hengduan Mountains and central China, generally to the east of the *Cesavelia* distribution area (Fig. 6). The remaining species are continuously distributed within this geographical range.

Color patterns and the shape of the connexival spines exhibit considerable intraspecific diversity (Tran et al. 2009; Jin et al. 2023), and do not appear to be reliable indicators for assessing phylogenetic relationships. We have observed a high species diversity of *Cesavelia* in the Himalayan and Hengduan Mountain regions. The reconstruction of the phylogenetic relationships within this subgenus, along with an in-depth investigation into how the Himalayas and Hengduan Mountains have influenced its speciation, dispersal, and diversification, warrants further study.

### ﻿Species list of Velia (Cesavelia) recorded in China

*V. sinensis* Sichuan Province

*V. tonkina* Yunnan Province

*V. yunnana* Yunnan Province

*V. longiconnexiva* Guizhou Province

*V. bui* Hubei Province

*V. lii* sp. nov. Xizang Province

*V. motuoensis* sp. nov. Xizang Province

*V. yiliangensis* sp. nov. Yunnan Province

## Supplementary Material

XML Treatment for Cesavelia

XML Treatment for Velia (Cesavelia) lii

XML Treatment for Velia (Cesavelia) motuoensis

XML Treatment for Velia (Cesavelia) yiliangensis

## ﻿Additional information

### Use of AI

No use of AI was reported.

### Author ORCIDs

Siying Fu https://orcid.org/0000-0001-7010-4401

Zezhong Jin https://orcid.org/0000-0001-7223-770X

Zhen Ye https://orcid.org/0000-0003-2327-1869

### Data availability

All of the data that support the findings of this study are available in the main text.
